# Antenatal and obstetric care in Afghanistan – a qualitative study among health care receivers and health care providers

**DOI:** 10.1186/1472-6963-13-166

**Published:** 2013-05-06

**Authors:** Zuhal Rahmani, Mette Brekke

**Affiliations:** 1 Faculty of Medicine, University of Oslo, Norway, Blindern, P.O. Box 1078, Oslo, 0316, Norway; 2 Department of General Practice, Institute of Health and Society, University of Oslo, P.O. Box 1130, Blindern, Oslo, 0318, Norway

**Keywords:** Antenatal care, Obstetric care, Afghanistan, Qualitative study

## Abstract

**Background:**

Despite attempts from the government to improve ante- and perinatal care, Afghanistan has once again been labeled “the worst country in which to be a mom” in Save the Children’s World’s Mothers’ Report. This study investigated how pregnant women and health care providers experience the existing antenatal and obstetric health care situation in Afghanistan.

**Methods:**

Data were obtained through one-to-one semi-structured interviews of 27 individuals, including 12 women who were pregnant or had recently given birth, seven doctors, five midwives, and three traditional birth attendants. The interviews were carried out in Kabul and the village of Ramak in Ghazni Province. Interviews were taped, transcribed, and analyzed according to the principles of Giorgi’s phenomenological analysis.

**Results:**

Antenatal care was reported to be underused, even when available. Several obstacles were identified, including a lack of knowledge regarding the importance of antenatal care among the women and their families, financial difficulties, and transportation problems. The women also reported significant dissatisfaction with the attitudes and behavior of health personnel, which included instances of verbal and physical abuse. According to the health professionals, poor working conditions, low salaries, and high stress levels contributed to this matter. Personal contacts inside the hospital were considered necessary for receiving high quality care, and bribery was customary. Despite these serious concerns, the women expressed gratitude for having even limited access to health care, especially treatment provided by a female doctor. Health professionals were proud of their work and enjoyed the opportunity to help their community.

**Conclusion:**

This study identified several obstacles which must be addressed to improve reproductive health in Afghanistan. There was limited understanding of the importance of antenatal care and a lack of family support. Financial and transportation problems led to underuse of available care, especially by poorly educated rural women. Patients frequently complained of being treated disrespectfully, and health care providers correspondingly complained about poor working conditions leading to exhaustion and a lack of compassion. Widespread corruption, including the necessity of personal contacts inside hospitals, was also emphasized as an obstacle to equitable antenatal and obstetric health care.

## Background

Although maternal mortality in Afghanistan has declined from around 1000 to 460 per 100 000 live births between 2000 and 2010 [1], the lifetime risk of dying in childbirth is still one in 32, comparable to conditions in Sub-Saharan Africa [2]. In addition, for each woman who dies, 20 others are expected to endure long-term ill health [3]. According to Save the Children’s World’s Mothers’ Report, Afghanistan is the “the worst country in which to be a mom” and one out of five children die before their fifth birthday [4].

According to the WHO, skilled health personnel attend approximately 34% of births in Afghanistan, and 60% of pregnant women attend at least one antenatal visit [1]. Only 16% attend the recommended number of four antenatal visits [1] and conditions are far worse in remote districts compared to Kabul and other main cities [5]. A survey from 2006 showed that more than half of women in Kabul had access to midwifery care, compared to less than 2% of women in remote areas [6].

Development in Afghanistan is complicated by an unstable political system, poor economy, and ongoing violence [7]. These factors severely influence health care provision and the quality of health care. The governmental guidelines for reproductive health care recommend four antenatal visits which are free of charge in public clinics, in an attempt to increase skilled attendance at birth, while improving quality and utilization of emergency obstetric care [8]. Despite these good intentions, such guidelines are unrealistic given the low density of physicians (2.1 per 10 000 inhabitants) and nursing and midwifery personnel (5.0 per 10 000 inhabitants) [1], with a strong urban–rural disaggregation of health worker density [6].

The aim of the present study was to explore how pregnant women and health care providers experience the existing antenatal and obstetric health care situation in Afghanistan. Rather than further compile poor statistics on reproductive health and health care in Afghanistan, we opted for a qualitative approach to explore the perspectives of patients and health professionals. The first author was born in Afghanistan and is a medical student in Norway – “the world’s best country in which to be a mom” [4]. A PubMed search using the keywords “antenatal care” or “obstetric care” or “reproductive health” and “Afghanistan” (May 2010) identified a paucity of research, with only a handful of relevant studies. A survey carried out in 2004 found reproductive health indicators to be poor among women living in Kabul, a group often considered to be the most privileged [9]. The women’s schooling was significantly associated with antenatal care attendance, skilled attendance at birth and use of family planning, although almost all the women needed permission from their husband or a male relative before seeking professional health care [9]. A cross-sectional study covering almost 5000 women in the Herat province found that human rights factors contributed to the high maternal mortality rate, particularly among rural women [10]. Another survey from Kabul revealed that fear of own death or of losing the baby were important reasons for choosing skilled birth attendance [11]. A 2003 observational study from a maternal and infant hospital in Kabul concluded that profound changes were needed in the hospital’s health care delivery system in order to make the hospital a safe and effective health care facility [12]. To expand upon this literature and improve our knowledge regarding obstacles to care, we chose to carry out the present study.

## Methods

### Study design

This study utilized a qualitative design based on semi-structured, one-to-one interviews. The interviewer (first author) spent several days in each clinic, closely observing the work of the health personnel and the facilities and services available to patients.

### Ethics

The study was approved by the Regional Ethics Committee for Medical Research in Southeastern Norway. In addition, the Ministry of Health in Kabul provided written permission to observe the health care facilities and conduct the interviews. Participants received written information about the study and the role of the interviewer and provided written consent. For participants who were illiterate, oral information was provided and verbal consent was obtained. All information and materials were provided in the Dari language.

### Participants

A total of 27 persons were interviewed. These included 12 patients (pregnant women or women who had recently given birth), seven doctors, five midwives and three traditional birth attendants (Table 1). No interview appointments had been scheduled in advance, and thus, interviews were arranged impromptu on-site. Participants were invited to participate in the study if time and other situational factors allowed. Individuals who agreed and provided informed consent were interviewed immediately without delay. Of those who were approached, ten patients refused participation in the study, while all invited health care providers agreed to participate.

**Table 1:** Interview location of the 27 participating patients and health care providers

***Kabul***			***Ghazni (Ramak)***
***Malalai Maternity Hospital***	***Ali Sina Clinic***	***Marie Stopes Int. . Clinic***	
6 patients (P 1–6)	1 patient (P 7)	1 patient (P 8)	4 patients (P 9–12)
4 doctors (D 1–4)	1 doctor (D 5)	1 doctor (D 6)	1 doctor (D 7)
5 midwives (M 1–5)			
			3 traditional birth attendants (TBA 1–3)

P = patient; D = doctor; M = midwife; TBA = traditional birth attendant.

### Setting

The interviews took place during the summer of 2010 in two different provinces in Afghanistan: Kabul and Ghazni. In Kabul, the locations were Malalai Maternity Hospital (6 patients, 4 doctors, 5 midwives), Ali Sina Clinic (one patient, one doctor) and Marie Stopes International Clinic (one patient, one doctor). Malalai Maternity Hospital is the largest women’s hospital in Afghanistan, with 80–100 births daily. Ali Sina Clinic is a private clinic providing obstetric services as well as internal medicine and surgery. Marie Stopes International Clinic belongs to a UK-based, non-governmental organization and provides mainly reproductive health services. In Ghazni (143 kilometers southeast of Kabul), the interviews were carried out in the village of Ramak, 30 km from the center of Ghazni, either in the village’s only clinic or in the participants’ home (4 patients, one doctor, 3 birth attendants).

### Questionnaires

Two semi-structured questionnaires designed specifically for this study were used, one for patients and one for health care providers (Appendix: Questionnaires I and II). Patients were asked about education, parity, present or previous pregnancy complications, help-seeking behaviour, obstacles to receiving antenatal or obstetric care, sources of information regarding pregnancy, expectations and satisfaction regarding care, and how they had been received by health care providers. Doctors, midwives, and birth attendants were asked if they were familiar with national guidelines for antenatal care, if they adhered to these guidelines (or why not), their own views on the quality of care they were able to provide, and personal attitudes towards patients. They were also asked about areas needing improvement and personal views on reasons for treatment delay.

### Data collection

All interviews were carried out by the first author in Dari, which was the participants’ and the interviewer’s first language. Interviews were either taped or – in case of background noise – written down word for word.

### Data analysis

All collected data were translated into Norwegian by the first author. Data were analyzed according to the principles of Giorgi’s phenomenological analysis, modified by Malterud [13,14]. The analysis included four steps: 1) reading the material to obtain an overall impression, 2) identifying units of meaning representing the various topics explored, 3) condensing and grouping the units of meaning, and 4) describing various aspects of antenatal and obstetric care from the patients' and health care providers' perspectives based upon these groupings. In addition, observations made by the interviewer during the time spent in the clinics provided a background for interpreting the material.

## Results

### Characteristics of participants

The median age of patients (P) was 28 years (20 – 38 years). Four of the 12 women were literate, all of whom lived in Kabul. Parity varied from one to nine, with a median parity of 3 in Kabul and 8 in Ghazni. Eight women had experienced complications in the present or previous pregnancies.

The seven doctors (D) were 35 to 50 years of age; only one was male, and all claimed to be specialists in gynaecology/obstetrics. Afghanistan has five medical faculties, where students receive six years of education followed by one year of clinical residency before being granted authorization to practice medicine.

The five midwives (MW) were all women, similarly aged as the medical doctors. Midwifery in Afghanistan requires two years of theoretical and practical curriculum following the completion of high school.

The three traditional birth attendants (TBA) were women over the age of 50 with at least 15 years of clinical experience. All three birth attendants were illiterate. Two had been educated by NGOs to become “community health workers” and promoted family planning, improvements in hygiene, etc. The birth attendants supervised normal pregnancies and childbirths and encouraged the women to seek professional help if complications were suspected. They did not perform vaginal examinations.

### Interview data: main findings

Several units of meaning emerged from the data analyses. Notably, the patients reported underusing available antenatal care. Reasons for underuse of services included low motivation, family decisions, lack of money, or transportation problems. The women reported being treated poorly by health professionals, and that bribery or personal contacts were needed to secure good care. Despite poor working conditions, health care providers were proud of their job, and the women were grateful for the help available. Each specific unit of meaning is discussed in greater detail below.

1. Available care is underused

1.i. Motivation is low

National guidelines recommend at least four antenatal check-ups [8]. Only five of our 12 participants attended all four recommended checkups – two women attributed attendance due to complications. Notably, these five women reported no obstacles to seeking health care; they all lived in an urban area under reasonably good socioeconomic conditions and four were educated. The other seven women attended antenatal health care visits only if they felt unwell:

*No reason to seek health care when you feel healthy* (P 5).

*I see a doctor only when it is absolutely necessary, otherwise it is not worth the effort* (P 10).

*Rural people do not attend check-ups, we are used to managing on our own* (P 12).

These selected statements reflect limited insight regarding the importance of regular antenatal care. It also mirrors a low level of knowledge regarding pregnancy and childbirth – only two women said they had solicited information from health personnel. The others relied upon their own experiences or those of female friends.

1.iv. Transportation difficulties

In Ramak, transportation problems were identified as a primary obstacle to seeking health care. In rural Afghanistan, roads are poor and public transportation is mostly very limited. In Ramak, people may have to walk one to two hours to get to the local clinic, as there are no taxis or local buses. From the village center of Ramak, a bus goes to Ghazni three times a day, and not after dark. This crowded bus represents the only transportation option for pregnant women if they want to attend the district hospital in Ghazni. The women – wearing a burqa – do not feel comfortable in a crowded bus with unknown men, and their husbands are reluctant to let them travel. Furthermore, women experiencing complications during labor also have to rely on the bus to get to the hospital. At night, when the bus does not run and the local clinic is closed, transportation and accessibility issues imply serious challenges to receiving adequate care. Someone owning a vehicle has to be found and asked to drive the woman to hospital. This may be time-consuming and difficult, as very few in Ramak own a car.

*It is worst in the evening when the local clinic is closed and when there are serious complications. We are not always able to find a car to transport her to the hospital in Ghazni soon enough. We call on the only (male) local doctor and do our best to save mother and child, within our limits* (TBA 1).

1.iv. Money issues

Poor economic conditions are also emphasized as a main obstacle to seeking necessary medical care without delay:

*People cannot afford food, how can they pay for medications, not to mention travel expenses to see the doctor?* (TBA 3).

All doctors and midwives identified financial hardship as an important reason for health care underuse or treatment delay. Rural and urban women both confirmed that a lack of money was a primary factor affecting treatment seeking or treatment adherence:

*We are poor people. We cannot afford to go to the hospital in Ghazni where treatment is better. This requires a lot of money and is expensive. Instead we go to the local clinic (in Ramak) and hope that the doctor there can help us* (P 9).

*To see doctors and buy medications for my pregnancy complications was an economic burden to our family. Sometimes we could not afford the planned follow-up* (P 3).

1.iv. Family decisions

The decision-maker in the family – typically the mother-in-law or the husband – may not support the pregnant woman in seeking medical care, even if complications are suspected. The woman is expected to be strong and enduring:

*You are not the first woman on earth to give birth!* (mother-in-law, cited by a pregnant woman) – (P 10).

The pregnant woman may be reluctant to reveal her symptoms or worries:

*I do not dare to tell anybody unless it is really serious; it is embarrassing* (P 11).

Traditional birth attendants (TBA) may provide support to rural women who are in a vulnerable position:

*They tell us they worry about their own and the baby’s health, but no one at home cares* (TBA 1).

When complications worsen and become obvious, the family often uses traditional remedies first, thereby delaying contact with health services. Only after traditional remedies fail is the pregnant woman taken to hospital.

Midwives worry about the misinterpretation of grave symptoms:

*We see that women with convulsions are first taken to spiritual men, because the family is convinced that the woman is possessed by evil spirits* (MW 2).

Doctors therefore frequently face serious, life-threatening situations:

*Our patients frequently arrive in a bad clinical condition, unfortunately we are not always able to save them* (D 1).

2. Dissatisfaction with attitudes and behavior of health personnel

2.ii. Poor working conditions contribute to poor behavior

Overworked personnel, long hours, low salary and lack of infrastructure are factors identified by doctors to explain negative attitudes and unprofessional behavior:

*The majority of our employees are women. In addition to poor working conditions and high stress level at work they have to take care of their family and do housework. No wonder one gets exhausted and gets into conflicts with patients* (D 4).

One midwife:

*We are very busy here, 120-130 patients a day. The situation is intolerable, of course we get tired and burned out. We keep going because we need a job, but this threatens our health* (MW 1).

2.ii. Lack of professional ethical standards

In Afghanistan, physicians occupy an authoritarian position in society and their opinions and behavior are supposed to be accepted. The patient, in contrast, is positioned at the bottom of the hierarchy. In a typical consultation, the patient humbly reveals her symptoms, and is soon interrupted by the doctor and handed a prescription, without any explanation.

Nine of our patients said that health personnel had behaved in an unfriendly and unprofessional manner. Further, patients reported incidents of abuse by a doctor or a midwife, including harsh words, arrogant behavior, even physical violence such as being hit or pulled by the hair:

*When I was having my first child, I got hit because I screamed in pain* (P 2).

*They are not kind* (P 2-7, P 10-12).

*When we see the doctor, it is because we are sick and vulnerable, a little smile and friendly behavior would make a big difference, we would feel better and taken care of* (P 7).

One doctor also described prevailing negative attitudes towards patients in the hospital:

*I am sad to say that patients are afraid of us, they do not dare to ask questions. If I take good care of my patient, my colleagues ask if I am related to the patient or have received money from her* (D 4).

An experienced midwife confirmed:

*Doctors at the clinic are irresponsible and uncaring. They claim payment for services and medications that are free for the patients. They keep the clinic open only a couple of hours and the government does not monitor this situation. Once a woman who came for an antenatal check-up was humiliated by the doctor because she had so many children and the doctor had no more time for her* (MW 3).

The traditional birth attendants who sometimes accompany patients to the district hospital reported the same:

*I remember well a midwife yelling at a patient who obviously was in pain, telling her that if she had learned how to get pregnant, she would have to learn to tolerate a little pain* (TBA 3).

This way of treating patients is also widespread among unskilled hospital staff:

*If the doctors cannot behave themselves, why should they?* (patient, about hospital assistants – P 3).

Conditions may seem better in private clinics. A patient attending a private clinic reported:

*After my second pregnancy I wanted a break until the next pregnancy. I went to a public family planning clinic for contraception, but instead I was accused of keeping the doctor busy, because he would rather be at home painting his house. I had to leave the clinic without contraception, I got pregnant, I never went there again* (P 7).

3. Personal contacts secure good treatment

Patients who are related to or know someone with authority at the hospital, such as a doctor, receive better care and cut queues. Even if the specific doctor is absent, other doctors treat the patient well, in order to do their colleague a favour. Most patients complained about this form of inequality:

*If you do not know anybody in the hospital, you may be left to yourself and forgotten* (P 2).

Doctors and midwives confirmed this issue and said that women of low socioeconomic status from rural areas were particularly at risk.

4. Corruption - fraud or necessity?

Even if treatment at public clinics is intended to be free of cost, corruption is frequent, also at clinics offering perinatal care. Employees may demand payment for medications or contraceptives, which should be delivered for free:

*Rumors say that personnel at the clinic sell medications on the black market and tell patients that they have run out of medications* (P 10).

*It is common to pay bribes to hospital attendants to make them do as you wish* (woman who had recently given birth – P 6).

*In public clinics IUDs are supposed to b e free for patients. But if the patient does not pay the doctor, she gets no IUD* (D 4).

Low salary was cited as the reason for these customs, and health personnel claimed to depend upon the extra income to be able to support their families.

5. Satisfaction after all

Despite these serious concerns, the patients expressed gratitude that some antenatal and obstetric care existed at all. They were grateful that help was available if their life was in danger:

*It is better than nothing. Earlier there was no such clinic with a female doctor in this community* (P 10).

Health personnel reported feeling proud of having a useful role in society, at least to the degree possible given the circumstances:

*Even if our productivity and the quality of work are limited by a lack of equipment and medications, we as doctors are obliged to do our best to save mother and child. We love our job, and it feels good to be able to help people* (D 1).

## Discussion

The main aim of this study was to explore how pregnant women and health professionals experience the quality and provision of antenatal and obstetric care in Afghanistan. Using a qualitative approach, we explored patient perspectives regarding obstacles and challenges to safe and comprehensive care. We also assessed viewpoints and perspectives by health care providers, including physicians, midwives, and traditional birth attendants.

This study has several limitations that should be noted. A small number of individuals from only two selected geographical areas were interviewed. However, we attempted to include patients and providers from various health care settings: a big public hospital, a small village clinic, an NGO clinic, as well as a private hospital, to increase representativeness. Another potential limitation involves the translation process, as translating the interviews from Dari to Norwegian, and then publishing in English, may have led to inconsistencies. However, the first author is fluent in all three languages, the interviews were purposefully conducted using simple and unsophisticated language, and the item responses were easy to understand. Therefore, this potential limitation is considered to be of minimal importance. Additionally, the interviewer spent several days observing each health care facility, yet these observations were not systematized and incorporated into the study and this might be considered a weakness of the study. The role and position of the interviewer were made clear to all participants to avoid potential confusion.

Even if public antenatal care exists in Afghanistan and the government recommends routine check-ups, our study confirmed underuse of these resources. Our study identified numerous reasons why women may not utilize these accessible and available services. Participants reported several obstacles, including their own personal views and beliefs, family decisions, financial reasons, as well as transportation difficulties. The National Reproductive Health Strategy 2006–2009 document states that more than 70% of families in Afghanistan who did seek help encountered barriers either because of costs or poor quality offered at the only accessible health care facility [15]: “Health care knowledge and ability to seek care decline with remoteness and low literacy rate. Decisions to seek care are also influenced by the family’s perception of accessibility. Families do not seek care because of lack of transportation, insecure travelling conditions and inability to afford either transport or care.”

Insecurity, lack of infrastructure, economic instability, poor communication and coordination, poor public health infrastructure, as well as lack of qualified health workers are the main challenges to rebuilding the Afghan health care system [7]. Level of civil unrest was substantial when this study was carried out, especially in Ghazni. This may have also influenced the patients' perception of the accessibility of health care.

Barriers grounded in traditional belief systems that influence birthing generally or affect interpretation of complications are found to be important in several parts of the world, such as Bolivia, Jamaica, and Sub-Saharan Africa [16]. In our study, health personnel confirmed that this still is a substantial problem in Afghanistan, for example, serious complications may be interpreted as the woman being possessed by evil spirits. Social customs within an area may also influence women’s internal barriers, such as shame [17]. Feeling shameful or embarrassed by asking for help, from family members and health care workers, was stated by several of our participants.

In our study, patients reported being rejected at the clinic or were received in an unprofessional manner. This has also been found in other studies, mainly from countries in Latin America, Sub-Saharan Africa and South-Asia [16-20]. Maternity care can be disrespectful and inhumane, or even exploitative [17]. Offensive and demeaning language might be used by health personnel [18], as well as the carrying out of procedures during labour without information or discussion [19]. Women in a lower social position are considered particularly vulnerable to such negative experiences and this may certainly apply to women in Afghanistan. A 2008 study with focus groups of men or women in Afghanistan investigating the perception of quality in health services revealed that “poor people are treated badly in health services” [21]. A review from Vietnam suggested this may be partly mediated by behaviors of health care personnel who are likely to come from another social class and are unable to separate their discriminatory attitudes from their work as professionals [20]. Negative attitudes from health care staff may deter women from seeking health care, lead to treatment delay, and ultimately prevent good practice [20-23].

Socioeconomic inequalities in maternity care are huge in developing countries, and skilled birth-attendance is the least equitable intervention, due to problems with availability, accessibility, and affordability [24,25]. Most births without professional delivery care take place among the rural poor [6,24]. Those few women in our study who attended routine check-ups and reported no obstacles for seeking health care were educated and lived in an urban area under reasonably good socioeconomic conditions. A survey from 2006 showed that more than half of the women in Kabul had access to midwifery care, compared to less than 2% of women in remote areas. Poverty, low educational level, and more than two hours of travel were predictors for not seeing a midwife [6]. Maternal education has long been considered an important determinant for maternal and child health. Education implies increased knowledge about the health care system, as well as about danger signs and disease patterns [26,27].

All our participants reported the availability of at least some midwifery services, but the need for midwives in rural areas is still far from being met [1,6]. Afghan societal norms dictate that women should provide health care for women. During the Taliban period, virtually no female doctors or nurses were trained. Today, access to female health care workers is improving, but there are still many obstacles to overcome [7]. Studies from other countries, i.e. Vietnam, found that lack of privacy and insufficiency of female staff prevented women from seeking reproductive health care [16]. Under such circumstances, using the service of traditional birth attendants may be a reasonable choice. In a recent meta-analysis, training and support of traditional birth attendants has been found to positively affect perinatal outcomes [28].

The costs of obstetrical care can also be an important barrier to the poor [29]. Even when these services are officially free of cost, hidden costs due to corruption may add up to a substantial amount. At the level of individuals and households, there is mounting evidence of the negative effects of corruption on health in low- and middle-income countries around the world [30]. The poor are disproportionately harmed by corruption, because they are less able to afford bribes or the use of private initiatives [31-35]. In our study, bribery was reported to be necessary in order to obtain health services which were intended to be free of charge. This mirrors the dominating position of corruption in the Afghani society. A report from 2010 reveals that one third of the population sees corruption as a necessity, and up to 20% had paid bribes to a doctor or a lawyer during the last year [36]. A study from the Baltic States found that nearly half of those interviewed did not consider unofficial payments to health workers as corruption [33].

In a recent WHO and UNICEF report [37], Afghanistan was found to make some progress towards the Millennium Development Goal 5 - the reduction of maternal mortality - but insufficient progress towards Goal 4 – the reduction of mortality in children under five years of age, of which 40% of deaths occur during the first month of life. Our study has shed some light upon possible obstacles to achieving these improvements. Socioeconomic inequalities in the delivery of antenatal and obstetrics care are significantly greater than other forms of health care in developing countries [24]. Reducing these inequalities is essential if improvements in perinatal outcomes shall be reached, and this requires increased coverage among the rural poor [24]. These inequalities also mirror demand factors, and interventions aimed at overcoming barriers at the individual and community level have shown some positive effects in other developing countries [16,38]. For example, local women’s groups have been shown to affect birth outcomes positively in rural Nepal [38]. Improving the quality of care, including the behavior and attitudes of health personnel, and implementing effective systems for referral are important [16,39].

Medical education in developing countries should focus more on patient-centered care, including communication skills and attitudes [40]. Introduction of institutional and individual codes of conduct and continuous training of anti-corruption awareness and behavior are essential for health care providers [20,40]. Higher benefits for health care workers could also be an important step.

What works to improve perinatal health care in resource-poor settings is now clearer, but implementation remains a challenge [41,42]. It has been stated that underevaluating political issues and excessive belief in management are reasons why improvement in reproductive health has stalled [43]. Many countries – like Afghanistan – now have the political will to improve mother and child health care [15]. But still, “the availability of good medical care tends to vary inversely with the need for it in the population served” [44]. “Proportionate universalism” has been launched as a solution. This means that those in greater need ought to be treated in proportion to that greater need [45]. Reproductive health does not exist in a vacuum, it is affected by how society is organized. Gender inequality leading to early marriage and recurrent child-bearing, as well as limited education regarding sexual health are important barriers to progress [46]. In a wider context, a profound political movement is needed to put an end to gender oppression in order to create a better and more equitable world for women [47].

## Conclusion

Underuse of available antenatal and obstetric health care was attributable to a variety of factors, including a limited understanding of its importance to maternal and infant health, lack of family support, financial problems, and transportation difficulties, especially for poorly educated rural women. Patients frequently complained of being treated disrespectfully, and health care providers correspondingly complained about poor working conditions leading to exhaustion and lack of compassion. Widespread corruption, including the importance of personal contacts, was also emphasized as an obstacle to equitable antenatal and obstetric health care in Afghanistan.

## Appendix

### Questionnaire I

Patient Questionnaire

•Age

•Years of education

•Number of previous pregnancies. Any complications?

•Have you attended antenatal check-ups regularly in this pregnancy, or only when you felt the need for it?

•Who has carried out your check-ups?

•Any obstacles to attending check-ups?

•Who has provided information regarding pregnancy and childbirth? (Health professionals, friends, relatives, media, etc.)

•Have you found the information sufficient and satisfactory?

•Do you feel confident you will receive the care you need in your pregnancy?

•How do you feel about the care you receive here? (in this clinic)

•What are your experiences with the attitudes and behaviour of the health personnel?

### Questionnaire II

Health Care Provider Questionnaire (i.e., doctors, midwives, traditional birth attendants)

•Do you know if there are national guidelines for antenatal care?

•Do you follow these guidelines?

•If not – why not?

•What do you think of the quality of the care you are able to provide (in this clinic)?

•How do you evaluate the attitude of health personnel towards the patients (in this clinic)?

•What is needed to further improve ante-and perinatal care?

•What are the reasons that some pregnant women seek help too late?

## Competing interests

The authors declare that they have no competing interests.

## Authors’ contribution

ZR and MB planned the study together. ZR carried out the interviews, and also carried out the data analyses, with help from MB. The two authors drafted the manuscript together and both approved the final manuscript.

## Pre-publication history

The pre-publication history for this paper can be accessed here:

http://www.biomedcentral.com/1472-6963/13/166/prepub
